# Insight into the development of versatile dentin bonding agents to increase the durability of the bonding interface

**DOI:** 10.3389/fdmed.2023.1127368

**Published:** 2023-02-16

**Authors:** Isabel Cristina Celerino de Moraes Porto, Teresa de Lisieux Guedes Ferreira Lôbo, Raphaela Farias Rodrigues, Rodrigo Barros Esteves Lins, Marcos Aurélio Bomfim da Silva

**Affiliations:** ^1^ Laboratory of Characterization and Analysis of Biomaterials, Faculty of Dentistry, Federal University of Alagoas, Brazil; ^2^ Laboratory of Quality Control of Drugs, Medicines, Foods and Biomaterials, Institute of Pharmaceutical Sciences, Federal University of Alagoas, Brazil

**Keywords:** dental materials, dentin adhesion, hybrid layer degradation, dental adhesive bonding formulations, bonding agents

## Abstract

Despite the huge improvements made in adhesive technology over the past 50 years, there are still some unresolved issues regarding the durability of the adhesive interface. A complete sealing of the interface between the resin and the dentin substrate remains difficult to achieve, and it is doubtful whether an optimal interdiffusion of the adhesive system within the demineralized collagen framework can be produced in a complete and homogeneous way. In fact, it is suggested that hydrolytic degradation, combined with the action of dentin matrix enzymes, destabilizes the tooth-adhesive bond and disrupts the unprotected collagen fibrils. While a sufficient resin–dentin adhesion is usually achieved immediately, bonding efficiency declines over time. Thus, here, a review will be carried out through a bibliographic survey of scientific articles published in the last few years to present strategies that have been proposed to improve and/or develop new adhesive systems that can help prevent degradation at the adhesive interface. It will specially focus on new clinical techniques or new materials with characteristics that contribute to increasing the durability of adhesive restorations and avoiding the recurrent replacement restorative cycle and the consequent increase in damage to the tooth.

## Introduction

To achieve an effective and complete marginal sealing and clinical longevity, an ideal tooth–restoration interface must have long-lasting durability. In enamel, adhesion is formed by resinous monomers that polymerize in demineralized areas, creating a more stable and stronger adhesive interface. The durability of the resin–dentin bond depends on the quality of a microinterface, the so-called hybrid layer, which is achieved by the infiltration and polymerization of resinous monomers of the adhesive system through a network of collagen fibrils exposed by the action of acids on the dentin surface and into dentinal tubules and, consequently, by the resistance to degradation of each one of the bond components. While the enamel is a dry substrate, with hydroxyapatite (92% v.) being the main component of its mineral phase, the dentin is the opposite, which is a complex and wet substrate with an average 45% (v) of hydroxyapatite crystals, 33% (v.) of organic matrix, mostly collagen type I, and 22% of water by volume (1, 2).

Although the exact mechanism responsible for hybrid layer degradation has not been completely understood, this process is thought to involve the hydrolytic degradation of monomers in water-rich zones within the hybrid layer, followed by enzymatic action on exposed collagen fibrils with relevant implications for the maintenance of the integrity of the adhesive interface (3). Many attempts to develop new functional adhesives that limit enzymatic degradation and polymer damage and have antimicrobial action have been presented (1, 4). Some strategies to modify the dentin surface, such as reducing the remaining demineralized collagen and applying ethanol to reduce water, seem to help increase the immediate bond strength. However, no progress has been made toward a synthesis of new materials as well as improving or modifying techniques in order to achieve good long-term results.

This review discusses recent clinical techniques and materials with characteristics that contribute to increasing the durability of adhesive restorations. The objective of this study is to present strategies that have been proposed in recent years for the improvement and/or development of new adhesive systems that can help prevent degradation at the adhesive interface.

## Matrix metalloproteinase inhibitors and collagen cross-linking agents

Matrix metalloproteinases (MMPs) are a group of more than 25 secreted and membrane-bound enzymes responsible for damage to the pericellular substrate. The main organic component of the tooth structure is collagen, and MMPs that degrade collagen and the extracellular matrix have been held responsible for the degradation of the hybrid layer (5).

The organic constituents of the hybrid layer, especially collagen, undergo degradation because of the enzymatic activity of MMPs, among other proteases present in the dentin matrix (6, 7). When the pH of the environment decreases to 4.5 or less, such as during the carious process and during the acid etching of the dentin surface that is carried out during the adhesive procedure, the MMPs that are in a latent state are activated and their action triggered by way of degrading the collagen that may have remained exposed underneath the formed hybrid layer. On the other hand, the acidic adhesive systems can also induce the activation of MMPs in the dentinal substrate. Thus, even if there is an inhibition of a part of the MMPs exposed by the demineralization of the dentin tissue, other MMPs would be activated in the presence of acidic adhesives (6, 8).

Once activated, collagenases cleave triple helical collagen molecules into two fragments of one-quarter and three-quarter length. The first fragment of one-quarter contains the C-terminal part of the collagen molecule and the second one of three-quarter contains the N-terminal portion (9). Collagen loses its triple helical conformation and can then be degraded by gelatinases MMP-2 and MMP-9 (10). MMP-2 and MMP-9 damage the non-helical fragments and reduce them to even smaller peptide fragments. The binding sites of collagen to MMPs are very similar to the catalytic sites of these enzymes. Thus, the collagen-binding domain of MMP-2 and MMP-9 binds to a short segment of the collagen *α*1 chain (11). After binding to the substrate, the water molecule that is bound to the zinc of the catalytic domain attacks the substrate and, through the transfer of protons, promotes a cleavage of the peptides (12). Endogenous MMPs in the dentin matrix are presumed to bind to collagen peptides via their hemopexin-like domains (MMP-1, MMP-8) and/or their fibronectin-like domains (MMP-2, MMP-9) (13).

Studies have been carried out with several substances in an attempt to inhibit MMPs, aiming to improve the durability of the adhesive interface (14). However, issues such as the substantivity of MMP inhibitors and their release from polymer matrices remain (15). Synthetic or natural bioactive agents that inhibit the endogenous enzymes of the organic collagen matrix have been used in an attempt to produce a strong and durable bonding interface (14), and chlorhexidine (CHX) is the most widely used (16). These compounds can be applied in different ways; for example, they can be incorporated into the acid-etching gel, added into the adhesive system, or applied directly on the dentin surface after acid etching, while remaining in contact with the dentin surface (14, 17–27).

MMP inhibitors act through different mechanisms such as by chelating cations (17, 28, 29), collagen cross-linking (30–32), and competitive inhibition for active sites of the collagen molecule (12, 33, 34). In Table 1, which is modified from de Moraes et al. (35), different techniques and materials using various types of MMP inhibitors and their mechanisms of action are presented. Many studies have applied CHX as a potential inhibitor of MMPs (36–45). CHX can interact with the organic components of the dentin matrix. This interaction occurs through electrostatic forces, between CHX-protonated amine groups, mineral phosphates, and non-collagenous phosphoproteins, which, in turn, are closely related to collagen fibrils (46). The preservation of collagen integrity in the hybrid layer occurs through the interaction of CHX with the sulfhydryl group on the domain or cysteine located at the active site of the MMPs. This may distort MMP molecules and prevent them from binding to substrates, thus competing for the binding of calcium and zinc ions to MMPs because of their chelating action. Without these ions, MMPs will lose their catalytic activities (47). *In vitro* studies have shown that CHX can preserve the bond durability of adhesives up to 12 months (37, 38, 44). Nonetheless, the clinical trials carried out by Araújo et al. (41), Sartori et al. (42), and Valério et al. (40) concluded, after a 2-, 3-, and 4-year follow-up, respectively, that the addition of CHX into the primer of the self-etch adhesive or the application of CHX on the etched dentin did not provide any clinical advantages nor did it improve the clinical durability of adhesive restorations, compared with the groups without CHX.

**Table 1:** Matrix metalloproteinases inhibitors and strategies to improve the stability of the dentin/resin bonding [Moraes et al (35)]^a^.

Mechanism of action	MMP inhibitor	Method of use	Period of testing	Type of study	References
Chelation	CHX	2% diacetate CHX added into an experimental adhesive	1 year	*In vitro*	Da Silva et al*.* (36)
		2% digluconate CHX, dentin pretreatment, 60 s	6 months	*In vitro*	Lenzi et al*.* (37)
		0.01%, 0.05%, 0.1%, and 0.2% diacetate CHX added into a commercial adhesive	1 year	*In vitro*	Stannislawczuk et al*.* (38)
		0.5%, 1.0%, 0.2%, and 0.4% diacetate CHX into an experimental adhesive	18 months	*In vitro*	Menezes et al*.* (22)
		2% digluconate CHX, dentin pretreatment, 60 s	20 months	Clinical trial	Ricci et al*.* (39)
		2% digluconate CHX, dentin pretreatment, 30 s	1 year	Clinical trial *In vitro*	Brackett et al*.* (27)
		2% digluconate CHX, dentin pretreatment, 60 s	4 years	Clinical trial	Valério et al*.* (40)
		1% digluconate CHX added into a commercial self-etch adhesive primer	2 years	Clinical trial	Araújo et al*.* (41)
		2% digluconate CHX, dentin pretreatment, 30 s	3 years	Clinical trial	Sartori et al*.* (42)
		2% digluconate CHX, dentin pretreatment, 60 s	1 year	Clinical trial	Galafassi et al*.* (43)
		2% digluconate CHX, dentin pretreatment, 20 s	1 year	*In vitro*	Daood et al*.* (44)
		2% digluconate CHX, dentin pretreatment, 60 s	1 year	*In vitro*	Tekçe et al*.* (45)
	EDTA	EDTA 0.5 M, dentin pretreatment, 60 s	1 year	*In vitro*	Tekçe et al*.* (45)
	Tetracyclines	2% minocycline, dentin pretreatment, 60 s	6 months	*In vitro*	Singh et al. (46)
	Zinc	ZnO or ZnCl_2_ added into an experimental adhesive and primer	24 h	*In vitro*	Osório et al*.* (34)
	Galardin	0.2 mM galardin, dentin pretreatment, 30 s	1 year	*In vitro*	Breschi et al*.* (30)
		5 μM galardin added into an experimental adhesive	1 year	*In vitro*	Da Silva et al*.* (36)
	Batmastati	5 μM batmastati added into an experimental adhesive	1 year	*In vitro*	Da Silva et al*.* (36)
	QAS	2%, 5%, and 10% QAS, dentin pretreatment, 2 min	14 days	*In vitro*	Daood et al*.* (44)
	Quaternary ammonium Methacrylates	5% MDPB, 30% METMAC, MCMS, MAPTAC, DDAC, ATA, dentin pretreatment	1 month	*In vitro*	Tezvergil-Mutluay et al*.* (47)
	BAC	BAC-containing acid etchant, 15 s	1 year	*In vitro*	Comba et al*.* (21)
		0.5% BAC and 1% methacrylate BAC, 30 min	6 months	*In vitro*	El-Gezawi et al*.* (19)
		1% BAC-containing acid etchant, 15 s 0.5% and 1.0% BAC-containing acid etchant, 15 s 0.5% and 1.0% BAC-adhesive	6 months	*In vitro*	Sabatini et al*.* (48)
		1% BAC, dentin pretreatment, 15 s	1 year	*In vitro*	Tekçe et al*.* (45)
	Alcohols	Methanol, ethanol, 1-propanol, 2-propanol, 1-butanol, 2-butanol, tert-butanol, 2-hydroxyethylmethacrylate, 1-pentanol, hexanols, heptanols, octanols, 1,2-ethanediol, and 1,3-propanediol	1 month	*In vitro*	Tezvergil-Mutluay et al*.* (49)
		A series of ethanol concentrations (50%, 70%, 80%, 95%, and 100%), 15 s each after acid etching	24 h	*In vivo* *In vitro*	Kuhn et al*.* (50)
Collagen cross-linking	PA	0.02%, 0.1%, and 0.5% PA, dentin pretreatment	12 h	*In vitro*	Hiraishi et al*.* (31)
		3.75% PA, dentin pretreatment, 10 s, 1 min, 30 min, 60 min, 120 min, 360 min, and 720 min	6 months	*In vitro*	Ephasinghe et al*.* (51)
		2% PA-containing acid etchant, 15 s.	6 months	*In vitro*	Hass et al*.* (18)
	GA	0.1% GA, dentin pretreatment, 60 s	24 h	*In vitro*	Chiang et al*.* (52)
		5% GA, dentin pretreatment, 60 s	24 h	*In vitro*	Cilli et al*.* (53)
		5% GA, dentin pretreatment, 60 s	6 months	*In vitro*	Li et al*.* (33)
		0.02%, 0.1%, and 0.5% GA, dentin pretreatment, 60 s	12 h	*In vitro*	Hiraishi et al*.* (31)
	RB	0.1% RB—1 min—UV; 0.1% RB—2 min—UV; 1.0% RB—1 min—UV, dentin pretreatment, 60 s	24 h	*In vitro*	Chiang et al*.* (52)
		0.1% and 1% RB—5 min—UV or light blue dentin pretreatment, 60 s	4 months	*In vitro*	Fawzy et al*.* (32)
	Resveratrol	100, 250, 500, and 1,000 *μ*g/mL resveratrol solution, dentin pretreatment, 60 s	4 months	*In vitro*	Porto et al*.* (25)
	Quercetin	5% quercetin added into an adhesive	6 months	*In vitro*	Gotti et al*.* (54)
		100, 250, 500, and 1,000 μg/mL quercetin solution, dentin pretreatment, 60 s	4 months	*In vitro*	Porto et al*.* (25)
Competitive inhibition for active sites of the collagen molecule	Zn	2% ZnCl_2_ or 10% ZnO added into a commercial etch and rinse adhesive 2% ZnCl_2_ added into a commercial self-etch primer adhesive 2% ZnCl_2_ or 10% ZnO added into a commercial self-etch bond adhesive	24 hours	*In vitro*	Osório et al*.* (34)
Multiple action mechanisms (by inactivating the active sites and improving the resistance of cross-linked collagen matrices)	Carbodiimides	0.01, 0.02, 0.05, 0.1, and 0.3 M carbodiimide, dentin pretreatment, 60 s	1 month	*In vitro*	Tezvergil-Mutluay et al*.* (13)
		1 M carbodiimide solution, dentin pretreatment, 60 s	6 months	*In vitro*	Singh et al*.* (46)
	EGCG Green Tea	0.1% EGCG solution, dentin pretreatment, 60 s	6 months	*In vitro*	Singh et al*.* (46)
		1.1% EGCG solution, dentin pretreatment, 60 s	6 months	*In vitro*	Monteiro et al*.* (55)
		0.1% EGCG solution, dentin pretreatment, 60 s	1 year	*In vitro*	Neri et al*.* (14)

MMPs, Matrix metalloproteinases; METMAC, [2(methacryloyloxy)ethyl] trimethylammonium chloride; MCMS, methacryloyl choline methyl sulfate; MAPTAC, [3(methacryloylamino)propyl] trimethylammonium chloride; DDAC, diallyldimethylammonium chloride; ATA, 2-acryloxyethyltrimethylammonium chloride; MDPB, 12-methacryloyloxydodecylpyridinium bromide; CHX, chlorhexidine; EDTA, ethylenediaminetetraacetic acid; QAS, quaternary ammonium silane; BAC, benzalkonium chloride; PA, proanthocyanidin; GA, glutaraldehyde; RB, riboflavin; EGCG, epigallocatechin-3-gallate.

^a^ With permission from authors.

Of late, other compounds have been presented, such as 2% doxycycline (56), 1,10-phenanthroline (57), captopril (58), ion-releasing filler (59), and *Emblica officinalis* (Indian gooseberry or amla) as an acid etchant and MMP inhibitor. Amla juice has a similar etching effect as a self-etch adhesive in SEM and 100% amla extract has been found to inhibit MMP-9 by gelatin zymography (60) and a novel mussel-inspired monomer [N-(3,4-dihydroxyphenethyl) methacrylamide] (61) with the aim of preserving the bonding between the restorative material and the tooth wall.

## Polymer hydrolysis

Although several studies consider adhesion to enamel to be a reliable approach, adhesion to dentin remains a crucial challenge (62). Differences in hydrophilicity and water content of adhesives directly influence the durability of interfaces. The presence of water plays an important role both in the prior acid-etching technique and in the self-etching technique. Water is an essential component in self-etching systems, as it enables the ionization of acidic monomers that demineralize the underlying enamel and/or dentin (63). In addition to having water in their composition, the ionizable groups of acidic monomers are hydrophilic. Therefore, different water sorption rates can be expected for the bonding interfaces produced by different bonding systems (64). Newer systems such as Universal or Multi-mode adhesives offer the option of selecting a conventional or self-etching bonding strategy, or an alternate strategy of “selective enamel etching” and self-etching into dentin. Despite the composition being similar to that of older self-etching adhesives, universal ones contain specific functional monomers of carboxylate and/or phosphate, with one of the main adhesives being 10-methacryloyloxydecyl dihydrogen phosphate (10-MDP), which ionically binds to dentin and is more effective and stable in water than that provided by other functional monomers (65).

There are several factors involved in the sorption of water and degradation of polymers, such as the following: the pH of the storage medium (66); conversion degree (67); the polarity of the molecular structure and the presence of hydroxyl groups able to form hydrogen bonds with water and the number of crosslinks (68); the presence of residual water and the presence and type of filler particles (69).

Hydrolysis is considered the main reason for resin degradation within the hybrid layer, contributing to a reduction in bond strength over time. After penetrating the polymer matrix, the water triggers the process of chemical degradation, resulting in the generation of oligomers and monomers. Water sorption and solubility rates presented by adhesive systems after polymerization are important for indirectly determining the longevity and marginal quality of the restoration (28). It has long been known that moisture present in the oral or storage environment plays an important role in the process of chemical degradation of polymers and has a deleterious effect on the resin–dentin interface (70).

Leaching is facilitated by the penetration of water into weakly cross-linked bonds or hydrophilic groups in the adhesive. Hydrophilic groups show limited monomer/polymer conversion because of adhesive phase separation (71) and a lack of compatibility between the hydrophobic photoinitiator and the hydrophilic phase (72). The poorly polymerized hydrophilic phase degrades rapidly in aqueous media (73). The structure of methacrylate adhesives, featuring hydrolytically susceptible groups such as ester and urethane, as well as hydroxyl, carboxyl, and phosphate groups (74), can be hydrolyzed by chemical and enzymatic degradation in an oral environment (75), and the collagen matrix previously infiltrated by resin becomes susceptible to attack by proteolytic enzymes (73).

Adhesive systems typically employ 2-bis[4-(2-hydroxy-3-methacryloxypropoxy)-phenyl]-propane (Bis-GMA) as the base monomer and low-viscosity 2-hydroxyethyl methacrylate (HEMA) as a monomer thinner to improve material quality and handling properties and to ensure proper dentin infiltration (76). However, adhesive durability and protective ability are often compromised by a failure of the tooth/restoration interface (77, 78). The issue of increasing durability and that of toxicity require the development of longer-lasting dental products without Bis-GMA/HEMA in adhesive systems.

New monomers and polymerization systems have been suggested to bypass the process of hydrolysis of the Bis-GMA/HEMA system (79), for example, triethylene glycol vinyl-benzyl-ether (TEGBE) monomer, which is a hydrolytically stable ether-based monomer (80). This compound has a dumbbell-shaped amphiphilic structure with a hydrophilic core and two hydrophobic vinyl-benzyl groups. Its polymerization mechanism does not show the limitation of diffusion and provides a higher degree of conversion (81). Song et al. (68). showed that the functional amino silane methacrylate monomer (ASMA) is able to act simultaneously as a co-monomer and co-initiator with the ability to develop a simplified adhesive that achieves greater durability and mechanical properties.

## Adhesive systems with antimicrobial activity

Adding antimicrobial substances to dental adhesives has been shown to improve tooth–restoration bonding (82), inhibiting the growth and multiplication of remaining bacteria (82, 83), while preventing the entry of new microorganisms and even limiting the influence of the factors that initiate the chemical and enzymatic degradation of dental adhesives (84). As a result, the durability of the tooth–restoration interface increases and there is a reduction in secondary caries.

The search for materials with antibacterial properties resulted in the development of new dental adhesives with antimicrobial agents, with inorganic fillers, modified monomers, or additives incorporated both in the polymeric matrix and in the filler particles (85–87) such as zinc oxide nanoparticles (88), silica nanoparticles functionalized with amphotericin B (89), silver nanoparticles (90–92), modified monomers containing quaternary ammonium (93), flavonoids (94, 95), plant extracts (94, 96), gold nanoparticles (97), calcium phosphate (87, 98), zinc oxide (99), and titanium dioxide (100, 101). Aguiar et al. (102) compiled various studies wherein commercially available antimicrobial adhesives (Clearfil™ SE Protect Bond/MDPB, Gluma 2 Bond/glutaraldehyde, Peak Universal Bond/chlorhexidine) and experimental materials or commercial adhesives modified with antimicrobial agents, including materials with quaternary ammonium methacrylate (QAM), dimethylamine dodecyl methacrylate (DMADDM), and dimethylamine hexa-decyl methacrylate (DMAHDM), silver nanoparticles (NAg), titanium dioxide (TiO_2_), zinc oxide (ZnO_2_)], silver- or zinc-doped bioactive active glass (BAG), titanium, and copper iodide, and compounds such as triclosan, quercetin, and grape seed extract, were analyzed. Compared with control groups, all these tested compounds showed the best results of inhibition for all tested pathogens, both single-layer bacteria and biofilm, without any adverse effects on the physicochemical and mechanical properties of adhesive systems.

Silver nanoparticles, either pure or mixed with other substances, were tested. The addition of silver nanoparticles in dental adhesives demonstrated specific antibacterial activity at the tooth–restoration interface (103, 104). In 2020, Münchow et al. (83) combined two processing methods (electrospinning and cryomilling) to obtain bioactive fillers based on fibers containing a strong MMP inhibitor, doxycycline, with antimicrobial and metalloproteinase inhibitor properties. The filler was used to obtain bioactive adhesives with the potential to inhibit MMPs and antibacterial activity against *S. mutans and Lactobacillus*, without compromising the physical–mechanical properties or the bond strength (up to 12 months).

Bacterial resistance against silver is difficult to achieve because of its multiple antibacterial mechanisms (105): (a) interaction with life-sustaining enzymes and blocking of the electron transport system in bacteria (106) and the thiol group of enzymes deactivating them, leading to bacterial cell death (107); (b) binding to bacterial cells by interacting with their cell membranes or cell walls. This can inhibit the movement of the organism or trigger leakage or rupture of the membrane (107, 108) (c) penetration inside the cell and the damage to intracellular structures (mitochondria, vacuoles, ribosomes) and biomolecules (protein, lipids, and DNA) (109); and (d) the formation of an organometallic complex when silver ions bind to the amino acids. Thus, silver ions can be generated inside the bacterial cell when this organometallic complex breaks down. The accumulation of silver ions in the cell can inactivate bacterial DNA and RNA, and it can damage and rupture the cell membrane, causing cell death (110).

## Bonding agents with the potential for biomimetic remineralization

To help remineralization of hard dental tissue is a desirable property in dentin bonding agents. Biomimetic remineralization is being considered a replacement for the restorative approach, which currently follows a minimally invasive procedure. Thus, selective caries tissue removal has become a clinical reality in deep cavities where demineralized dentin is left on the pulp areas and sealed with restoration (111).

In this sense, adhesion is a clinical challenge because conventional adhesives have low bond strength when applied to demineralized or contaminated dentin, as this dentin layer can be only partially infiltrated by resin monomers (112), becoming more prone to hydrolytic and/or enzymatic degradation, which may compromise the durability of composite resin restorations (113, 114).

Thus, the development of bioactive restorative materials capable of remineralizing mineral-depleted sites at the dentin–adhesive interface is one of the main aims of dental research (112, 115–117).

Studies using bioactive glasses incorporated in experimental adhesives provided mineral gain, increased microhardness, and better sealing of the resin–dentin interface (112, 115). The mechanism is based on the ability to release Si(OH)_4_ from the bioactive glasses, which binds to collagen exposed by acid etching and polymerizes into an absorbent SiO_2_-rich layer. This layer can aid in the precipitation of amorphous calcium phosphate (118) and subsequent conversion to nonstoichiometric apatite (119). In addition, a mineral-rich alkaline environment can allow a condensation of Si(OH)_4_ and precipitation of Ca^2+^ and PO_4_^−3^, and this can help fossilize metalloproteinases and cathepsins and reduce their proteolytic activity (28, 120).

It was also found that the use of materials with zinc can reduce metalloproteinase-mediated collagen degradation within poorly infiltrated hybrid layers and decayed dentin, protecting the sensitive collagen cleavage sites within demineralized dentin (121, 122), as zinc ions act as inhibitors of MMP-2 and MMP-9 (123).

The use of casein phosphopeptide-amorphous calcium phosphate (CPP-ACP) may be associated with an increased longevity of the dentin–resin interface (124–126) through the release, deposition, and stabilization of gradients with a high concentration of calcium and phosphate ions on the tooth (127).

The self-assembling peptide P_11_^−4^ (CH_3_CO-QQRFEWEFEQQ-NH_3_) was designed as a β-sheet-forming peptide that assembles into hierarchical structures in response to environmental triggers, resulting in the formation of a 3D biomimetic scaffold that is capable of nucleating hydroxyapatite, since the anionic groups present in P_11_^−4^ side chains attract calcium ions, inducing a hierarchical precipitation of calcium phosphate salts onto the preformed scaffold (128, 129). The self-assembled peptide possibly controls the deposition and growth of hydroxyapatite crystals on enamel (130–132). The behavior and performance of P_11_^−4^ in dentin has already been described (125, 126, 133) and may be a promising method in the treatment of caries-affected dentin, increasing the longevity of bonded restorations.

Sodium fluoride has also been shown to be a possible chemical inhibitor of MMPs (134, 135). However, Moreira et al. (126) did not detect an increase in bond strength when using sodium fluoride compared with CPP-ACP and peptide P_11_^−4^.

A complete remineralization of caries-affected dentin remains a challenge, but biomimetic remineralization strategies suggest that it is possible to promote mineral precipitation within the hybrid layer and reduce collagen degradation. However, more studies are needed to define viable clinical protocols and ascertain their long-term efficacy.

## Removal of residual water not bound to collagen

It is common knowledge that the adhesion of resin materials to conditioned enamel is significantly higher than that of dentin, which is mainly attributed to the characteristics of each substrate (136). The presence of water in the dentin hybrid layer during polymerization interferes with the quality of hybridization in this layer (137) and can cause hydrolytic degradation (138), a low-quality mechanical property (138), and nanoleakage (139).

Due to these problematics related to the presence of water in the hybrid layer, adhesive systems containing solvent and less hydrophilic monomers are preferred for residual water removal (139, 140). The types of solvent most commonly used in these systems are ethanol, acetone, and water. Ethanol binds to residual water through hydrogen bonds, increasing evaporation rates, and is more effective than water as a solvent. On the other hand, solvents containing acetone show promising performance in a simplified adhesive system, which uses a single bottle containing hydrophilic and hydrophobic monomers together (141). In addition, acetone contains a higher vapor pressure in addition to the water-chasing effect, which is believed to induce a greater removal of residual water (142). However, the application of both types of solvents in challenging clinical situations, as in non-carious cervical lesions, yields similar clinical performance and the same survival rates (143).

An alternate technique to remove residual water is called “ethanol wet bonding,” which is applied on etched and rinsed dentin, inducing the replacement of water by ethanol. In this way, ethanol carries out the process of chemical dehydration, increasing the interfibrillar spaces because of the additional interfibrillar shrinkage and reducing the hydrophilicity of the collagen matrix (139). However, it is necessary to point out that this technique is not a new one (144). Over a period of time, the scientific literature presents in vitro studies evaluating this technique, showing favorable and promising results (144, 145). However, when ethanol wet bonding is applied in in vivo studies or clinical trials, the results are not promising (50). Thus, the presence of sound dentin with a constant permeability through the dentinal tubules is a persistent challenge for the complete removal of residual water by ethanol (50, 147).

Other adhesive techniques with the same objective are proposed: the application of multiple adhesive layers or a layer of a hydrophobic resin agent and air-blowing the adhesive with high intensity are possibilities reported in the literature as viable (147). Likewise, a polar aprotic solvent, dimethyl sulfoxide (DMSO), was proposed as a component that improves adhesive infiltration and reduces residual water from the resin–dentin interface, thus improving the bond strength between resin-based materials and etched dentin (148).

It is relevant to emphasize the evaluation of adhesive systems and application techniques, from etch-and-rinse ones (by three or two steps) with a wet dentin substrate, as already mentioned, to a self-etch adhesive system (by two or single step) and a universal one, which allow working on a dry substrate, free from moisture (149, 150). Therefore, based on short-term randomized clinical trials, self-etch and etch-and-rinse adhesive systems applied with multiple coats appear to yield satisfactory clinical performance (149, 151). Also, as observed in a long-term clinical trial (13 years), according to a van Djiken study, a continuous and similar bonding degradation for all adhesive systems evaluated in non-carious cervical lesions was demonstrated (152).

## Conclusion and points of view for further research

Adhesive systems have improved over the years with regard to their interaction with the dental substrate, material composition, and technique. Even so, to increase the durability of the adhesive interface remains a challenge. The measures to improve the dental bonding systems presented in this review have been driven by the need to make the best use of the material to resolve issues such as enzymatic and hydrolytic degradation, preservation of the adhesive interface bonding strength over time, and the development of new bioactive products.

There is huge potential for the development of better, stronger, and bio-based materials purposely made from chemicals derived from renewable biological resources, such as natural polymers, propolis, vegetable oils, plant extracts, resins, and bioactive compounds. However, the accessed studies were done in a laboratory environment and in a short period of time. Even with excellent laboratorial results, the materials require further clinical investigation in different substrates (sound and carious dentin, abraded and sclerotic dentin) over a longer period of time in a challenging oral environment so that better results can be achieved and the presented evidence validated.
